# Supplementary material for: Metabolomics Approach Reveals Important Glioblastoma Plasma Biomarkers for Tumor Biology
Source: Int J Mol Sci. 2023 May 16;24(10):8813. doi: 10.3390/ijms24108813 (PMC10218163; doi:10.3390/ijms24108813)
Supplement: Supplementary file 1 [file ijms-24-08813-s001.zip › ijms-2306225-supplementary.pdf]

SUPPLEMENTARY MATERIAL

Title:

Metabolomics approach reveals important glioblastoma plasma biomarkers for tumor biology

Authors: Adriana C. Ferrasi[1], Ricardo Puttini[1], Aline F. Galvani[1], Pedro T. Hamamoto Filho[2], Jeany Delafiori[3], Victoria D. Argente[1], Arthur N. de Oliveira[3], Flávia L. Dias-Audibert[3], Rodrigo R. Catharino[3], Octavio C. Silva[1], Marco A. Zanini[2], Gabriel A. Kuorkawa[1], Estela O. Lima[1*]

1 Laboratory of Molecular Analysis and Neuro-oncology, Department of Internal Medicine, Botucatu Medical School, São Paulo State University, Botucatu, Brazil; adriana.ferrasi@unesp.br; ricardo.puttini@kroton.com.br; alinefgalvani@gmail.com; octavio.castro@unesp.br; estela.lima@unesp.br
2 Department of Neurology, Psychology and Psychiatry, Botucatu Medical School, São Paulo State University, Botucatu, Brazil; pedro.hamamoto@unesp.br; marco.a.zanini@unesp.br
3 Innovare Biomarkers Laboratory, School of Pharmaceutical Sciences, University of Campinas, Campinas, Brazil; jeanydelafiori@gmail.com; arthurnoin95@gmail.com; flaviald.nutricao@gmail.com; rrc@fcm.unicamp.br

* Correspondence: estela.lima@unesp.br; Tel.: +55 14 3880 1453

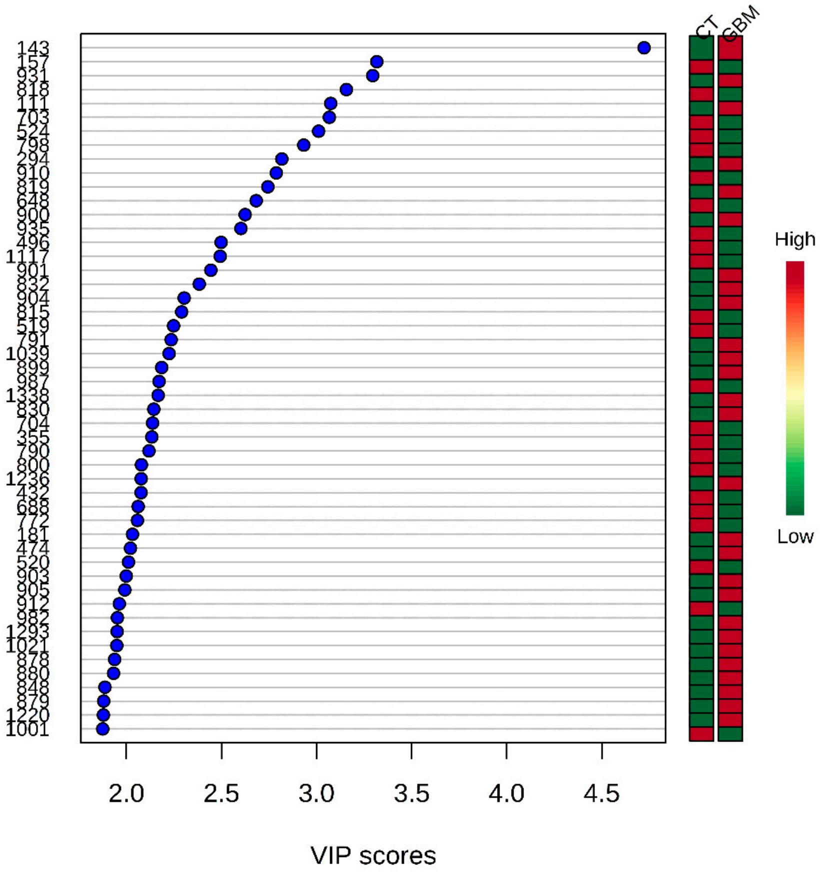

Figure S1. Variable Importance in Projection (VIP) plot with the top 50 most important features elected by PLS-DA for Control and Glioblastoma groups. The biomarkers behavior in each group is represented by the right columns, where red represents up-regulation and green, down-regulation.
